# Supplementary material for: Enhanced CH4 emissions from global wildfires likely due to undetected small fires
Source: Nat Commun. 2025 Jan 18;16:804. doi: 10.1038/s41467-025-56218-w (PMC11748658; doi:10.1038/s41467-025-56218-w)
Supplement: Supplementary file 1 — Supplementary Information [file 41467_2025_56218_MOESM1_ESM.pdf]

Supplementary information for:

Enhanced CH_4 emissions from global wildfires likely due to undetected small fires

Junri Zhao[1,2], Philippe Ciais[3,1], Frederic Chevallier[3], Josep G. Canadell[4], Ivar R. van der Velde[5,6], Emilio Chuvieco[7], Yang Chen[8], Qiang Zhang[9], Kebin He[2,10], Bo Zheng[1,2*]

[1]Shenzhen Key Laboratory of Ecological Remediation and Carbon Sequestration, Institute of Environment and Ecology, Tsinghua Shenzhen International Graduate School, Tsinghua University, Shenzhen 518055, China.

[2]State Environmental Protection Key Laboratory of Sources and Control of Air Pollution Complex, Beijing, 100084, China.

[3]Laboratoire des Sciences du Climat et de l’Environnement, LSCE/IPSL, CEA‐CNRS‐UVSQ, Université Paris-Saclay, Gif-sur-Yvette, France.

[4]CSIRO Environment, Canberra, ACT 2601, Australia.

[5]SRON Netherlands Institute for Space Research, Leiden, The Netherlands.

[6]Department of Earth Sciences, Vrije Universiteit, Amsterdam, The Netherlands.

[7]Universidad de Alcalá, Environmental Remote Sensing Research Group, Department of Geology, Geography and the Environment, 28801 Alcalá de Henares, Spain.

[8]Department of Earth System Science, University of California, Irvine, Irvine, CA 92697, USA.

[9]Ministry of Education Key Laboratory for Earth System Modeling, Department of Earth System Science, Tsinghua University, Beijing 100084, China.

[10]State Key Joint Laboratory of Environment Simulation and Pollution Control, School of Environment, Tsinghua University, Beijing, 100084, China.

*Corresponding author. Email: bozheng@sz.tsinghua.edu.cn.

Content of this file:

Supplementary Text 1

Supplementary Tables 1–3

Supplementary Figures 1–13

Supplementary Text 1: Methodology of atmospheric inversion

We employ the global 3-D transport model from Laboratoire de Météorologie Dynamique (LMDz), coupled with the Simplified Atmospheric Chemistry Assimilation System (SACS)[1], to simulate atmospheric CO dynamics in a 3-D grid which can be described by equation (1):

$$\begin{aligned}\frac{\partial[\mathrm{CO}]}{\partial \mathrm{t}} &= \sum(\mathrm{Source_{CO}}) - \mathrm{Sink_{CO}} \\ &= -\nu \cdot \nabla[\mathrm{CO}] + \sum_{sector}\left(E_{co}\right) + P_{\mathrm{CH_4 \to CO}} + P_{\mathrm{NMVOCs \to CO}} - k_{\mathrm{CO+OH}}(T)[\mathrm{CO}][\mathrm{OH}] - \mathrm{Dep_{CO}}\end{aligned} \tag{1}$$

The temporal evolution of CO concentrations (∂[CO]/∂t) in each grid cell is represented as the balance of CO emissions ($\mathrm{Source_{CO}}$) and CO removal ($\mathrm{Sink_{CO}}$). Where, the flux divergence term ($\upsilon \cdot \nabla$[CO]) accounts for CO transport across grid cells, which sums to zero globally. Surface emissions (E_{CO}) originate from sources like anthropogenic, biogenic, biomass burning, and oceanic sectors. Chemical production of CO from CH_4 and non-methane volatile organic compounds (NMVOCs), driven by OH oxidation ($P_{\mathrm{CH4 \to CO}}$ and $P_{\mathrm{NMVOCs \to CO}}$), is included, alongside CO's chemical sink ($k_{\mathrm{CO+OH}}(T)$[CO][OH]), which depends on temperature (T), CO concentration ([CO]), and OH concentration ([OH]). Dry deposition ($\mathrm{Dep_{CO}}$) contributes approximately 7% to the total CO sink[2].

The atmospheric Bayesian inversion framework is built upon the LMDz-SACS model[3,4], and satellite observations of relevant gases are assimilated to constrain the system[5-7]. The inversion inference is formulated as a variational optimization, minimizing the following cost function:

$$J(\mathrm{x}) = (x - x^b)^T \,\mathrm{B}^{-1}(x - x^b) + (H(x) - y)^T \mathrm{R}^{-1}(H(x) - y) \tag{2}$$

The control vector, x, contains the target variables, with x^b as the prior estimate, assuming Gaussian error statistics with covariance B. The observation vector, y, holds assimilated data, and its errors are also assumed Gaussian with covariance R. H is the forward model (a combination of the LMDz-SACS model, sampling operator, and averaging kernel) computes the observation equivalent from x. R incorporates

measurement, forward model, and representation errors. The inversion is solved iteratively using forward and adjoint codes until cost function convergence, yielding an optimized model state that fits all constraints within their uncertainties.

The inversion estimates emission fluxes in each grid cell with a horizontal resolution of 96 × 95 (1.9° latitude × 3.75° longitude) and a vertical resolution of 39 sigma-p hybrid levels. This off-line version of the LMDz model relies on pre-calculated air mass fluxes from the online version, which is nudged to the European Centre for Medium-Range Weather Forecasts (ECMWF) analysis for horizontal winds. The model incorporates direct, tangent-linear, and adjoint codes developed by Pison et al[1], based on the INCA full chemistry model. This framework simulates the hydrocarbon oxidation chain, including primary CO emissions, secondary CO production, and chemical sinks, using tracers such as CH_4, formaldehyde (HCHO), CO, and intermediate species, with all reactions driven by OH. The oxidation of CH_4 by OH initiates the chain, producing HCHO, which is further oxidized by OH to generate CO and H_2. The chain concludes with the oxidation of CO by OH to form CO_2. Additionally, the reaction of OH with methyl chloroform (MCF) is included in the inversion framework to constrain OH concentrations based on MCF measurements.

The modeling processes of the inversion system are outlined in Supplementary Fig. 13. This system infers surface flux variations of multiple species, including CO, CH_2O, CH_4, and MCF, by integrating atmospheric measurements of these tracers with the LMDz model and prior emission flux data. Since this study spans the period from 2003 onward, only CO observations were assimilated, as CO measurements are the only dataset available consistently from 2003. The priori emissions of CO, CH_2O, CH_4, and MCF serve as the initial input to the LMDz model and are transported at each simulated time step. Observation data for all tracers are compiled into an observation vector and used to evaluate model concentrations. The discrepancy between simulated and observed concentrations drives the inversion system to adjust the priori emissions, aligning the atmospheric observations with prior emission data while accounting for both of their uncertainties. To minimize errors arising from the initial state and the

prescribed OH field in the inverse modeling, the system is allowed to adjust the initial concentrations of tracers and OH during the assimilation period. Further details in the model process refer to our previous studies[5-7].

Supplementary Table 1. Annual global fire CH_4 emissions (Tg yr^{-1}) for the years between 2003 and 2020 derived from Fire Inventory from NCAR (FINN) v2.5[8], Global Fire Assimilation System (GFAS) v1.2[9], Quick Fire Emissions Dataset (QFED) v2.5r1[10], Global Fire Emissions Database (GFED) v4.1s[11], and our CO inversion-based fire CH_4 emission estimates. The uncertainty range for CO-based estimation is given in brackets.

	CO -based estimation	FINNv2.5	GFASv1.2	GFEDv4.1s	QFEDv2.5r1
2003	26.3 (6.3)	34.8	22.9	15.4	17.1
2004	27.3 (6.4)	34.5	21.7	15.6	16.8
2005	29.0 (6.6)	33.7	21.6	15.4	16.8
2006	25.2 (6.9)	29.8	22.7	19.6	15.3
2007	27.8 (6.0)	32.2	18.6	13.8	16.8
2008	21.4 (5.0)	25.4	17.6	11.6	14.9
2009	20.1 (5.4)	24.6	18.7	14.2	13.7
2010	26.1 (5.9)	28.8	16.7	13.9	15.1
2011	22.2 (5.3)	22.3	17.5	12.1	14.9
2012	24.3 (6.0)	26.2	21.7	14.3	15.7
2013	19.4 (5.4)	21.6	14.5	11.8	12.8
2014	24.1 (6.6)	23.7	20.8	16.7	13.6
2015	30.4 (8.4)	27.3	28.1	21.8	14.9
2016	23.8 (5.6)	23.8	17.2	12.1	13.6
2017	17.7 (4.4)	21.7	13.5	12.6	14.0
2018	18.5 (4.7)	20.6	14.9	12.5	12.7
2019	25.2 (6.9)	28.2	21.3	19.4	14.3
2020	23.2 (5.2)	26.5	12.9	12.9	13.9
Average	24.0 (5.9)	27.0	19.1	14.8	14.8

Supplementary Table 2. The 16 identified fire plume intercepts during ATom campaigns. The data listed in the table represents the minimum-maximum values for the respective dates. The observation datasets #1-8 were obtained from ATom 2, as identified in this study. #8-16 were obtained from ATom 3-4, as identified by Chen et al.,[12] which did not match biomass burning.

	Date	CH_4 (ppb)	CO (ppb)	HCN (ppt)	Biomas burning (%)	Fire inf (days)
#1	20170213	1884.1 – 1879.2	138.3 – 145.8	339.8 – 398.9	40.2 – 40.2	24 – 24
#2	20170213	1871.1 – 1874.8	140.2 – 147.8	366.2 – 436.5	42.9 – 44.7	16 – 17
#3	20170213	1844.6 – 1874.1	104.2 – 133.6	320.1 – 465.5	47.8 – 47.8	23 – 23
#4	20170213	1840.3 – 1885.7	100.4 – 166.4	320.7 – 465.5	56.7 – 88.6	12 – 21
#5	20170215	1840.6 – 1922.4	120.9 – 386.2	361.1 – 1108.5	67.3 – 86.2	6 – 23
#6	20170215	1857.5 – 1904.5	134.8 – 320.1	331.2 – 899.0	83.1 – 90.4	6 – 9
#7	20170215	1848.1 – 1920.3	120.5 – 416.3	331.0 – 1214.4	67.1 – 81.7	8 – 13
#8	20170215	1848.5 – 1920.3	120.0 – 218.4	331.1 – 782.2	58.6 – 63.7	13 – 21
#9	20171019	1871.5 – 1920.3	76.9 – 176.6	–	–	6 – 28
#10	20171019	1872.2 – 1905.4	73.5 – 116.1	–	–	5 – 29
#11	20171020	1872.2 – 1905.4	73.5 – 116.1	–	–	3 – 29
#12	20171020	1895.2 – 1908.6	91.8 – 149.6	–	–	15 – 26
#13	20180514	1843.9 – 1910.0	85.8 – 250.6	–	–	6 – 20
#14	20180514	1866.7 – 1910.1	99.9 – 150.4	–	–	3 – 13
#15	20180514	1869.6 – 1913.7	86.0 – 355.6	–	–	5 – 24
#16	20180514	1889.0 – 1893.7	86.1 – 113.9	–	–	21 – 22

Supplementary Table 3. Emission factors (EFs) (g kg^{-1}) for different fire types collected in this study. The standard error of the mean is given in brackets.

Source	EF CO	EF CH_4	Type
Vernooij et al.[13]	51(1.6)	0.74 (0.03)	Savanna
Vernooij et al.[13]	43 (2.0)	0.70 (0.04)	Savanna
Vernooij et al.[13]	46 (1.2)	0.69 (0.02)	Savanna
Vernooij et al.[13]	43 (3.7)	0.64 (0.06)	Savanna
Vernooij et al.[13]	52 (3.4)	0.90 (0.04)	Savanna
Vernooij et al.[13]	43 (5.3)	0.71 (0.10)	Savanna
Vernooij et al.[13]	138 (25.8)	5.18 (0.17)	Savanna
Frausto-Vicencio et al[14]	121 (12.2)	4.3 (0.8)	Temperate forest
Vernooij et al.[13]	80 (13.2)	2.06 (0.43)	Temperate forest
Wiggins et al.[15]	127 (40)	5.3 (1.8)	Boreal forest
Urbanski et al.[16]	78 (14)	2.14 (0.66)	Tropical forest
Urbanski et al.[16]	81 (18)	2.11 (0.77)	Tropical forest
Urbanski et al.[16]	85 (14)	3.32 (0.98)	Tropical forest
Urbanski et al.[16]	105 (20)	3.61 (1.05)	Tropical forest
Urbanski et al.[16]	53 (11)	1.39 (0.79)	Tropical forest
Yokelson et al.[17]	412.6 (24.5)	7.3 (0.3)	Peatland
Yokelson et al.[17]	361.7 (21.3)	7.8 (1.4)	Peatland
Yokelson et al.[17]	358.1 (11.3)	12.9 (1.2)	Peatland
Yokelson et al.[17]	339.6 (22.0)	9.7 (1.1)	Peatland
Lestari et al.[18]	236.5 (25.7)	5.43 (1.32)	Peatland
Hu et al.[19]	264	13.2	Peatland
Hu et al.[19]	41.7	9.2	Peatland
Watson et al.[20]	157 (24)	3.20 (0.69)	Peatland
Watson et al.[20]	159 (14)	6.94 (1.38)	Peatland
Watson et al.[20]	161 (19)	5.69 (1.07)	Peatland
Watson et al.[20]	394 (46)	10.42 (1.81)	Peatland
Watson et al.[20]	315 (10)	9.18 (0.26)	Peatland
Watson et al.[20]	93 (21)	7.65 (1.36)	Peatland
Watson et al.[20]	171 (22)	6.65 (0.93)	Peatland

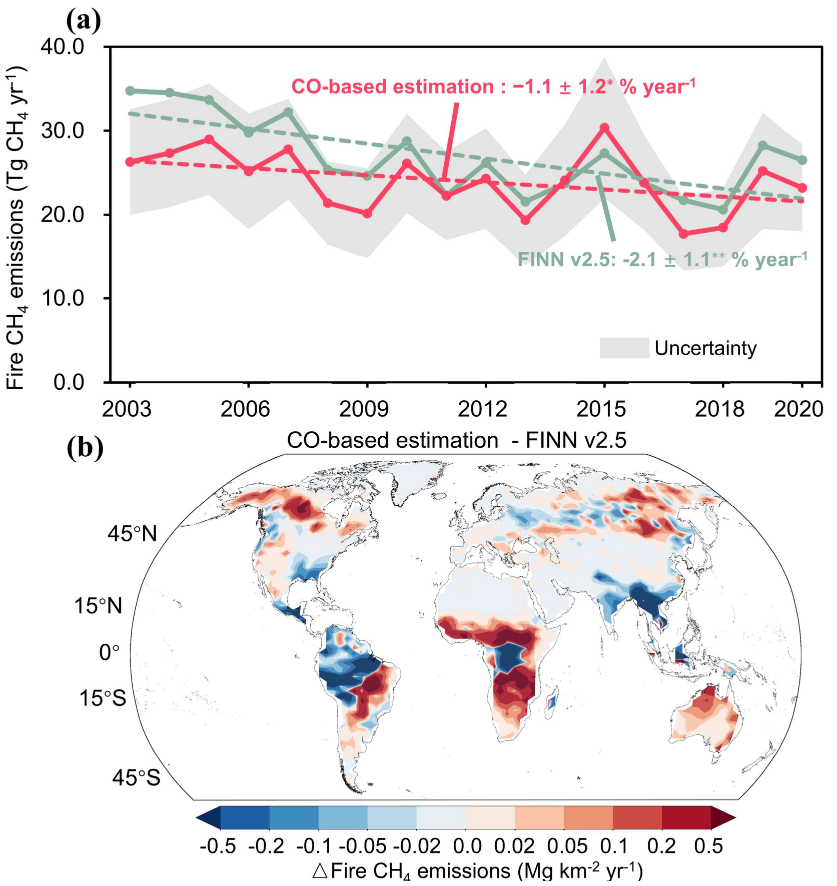

Supplementary Fig. 1: Comparison between global CO–based fire CH_4 emission estimates and FINNv2.5 results. (a) Annual trends in fire CH_4 emissions from CO–based estimates (red curve) and estimates of FINNv2.5 global fire emission model (green curve) from 2003 to 2020, including the fitted linear trends (dashed red and green lines). The shaded gray region represents the range of error resulting from uncertainties in CH_4/CO emission ratios and inversion-based CO estimates (Methods). Trend assessments are conducted using the nonparametric Mann–Kendall test and Theil–Sen estimator, with 2003–2020 trends and uncertainties provided. Significant trends are denoted by asterisks ($*p < 0.1$ and $** \ p < 0.05$). (b) Spatial distribution of differences between CO inversion–based CH_4 emission estimates and those of FINNv2.5. Data averaged between 2003 and 2020 are at the spatial resolution of 3.75° longitude × 1.9° latitude.

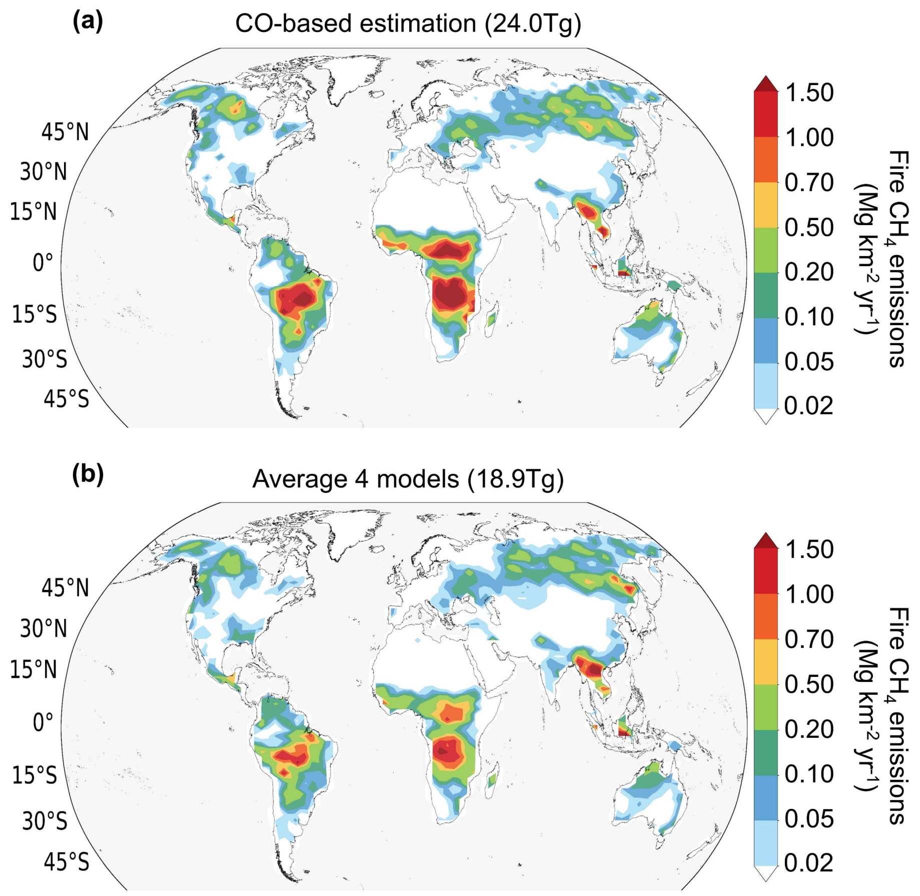

Supplementary Fig. 2: Spatial distributions of annual average fire CH_4 emissions between 2003 and 2020 derived from CO -based estimation (a) and bottom-up fire CH_4 emissions (b). The spatial resolution of emission maps shown here is 3.75° longitude ×1.9° latitude.

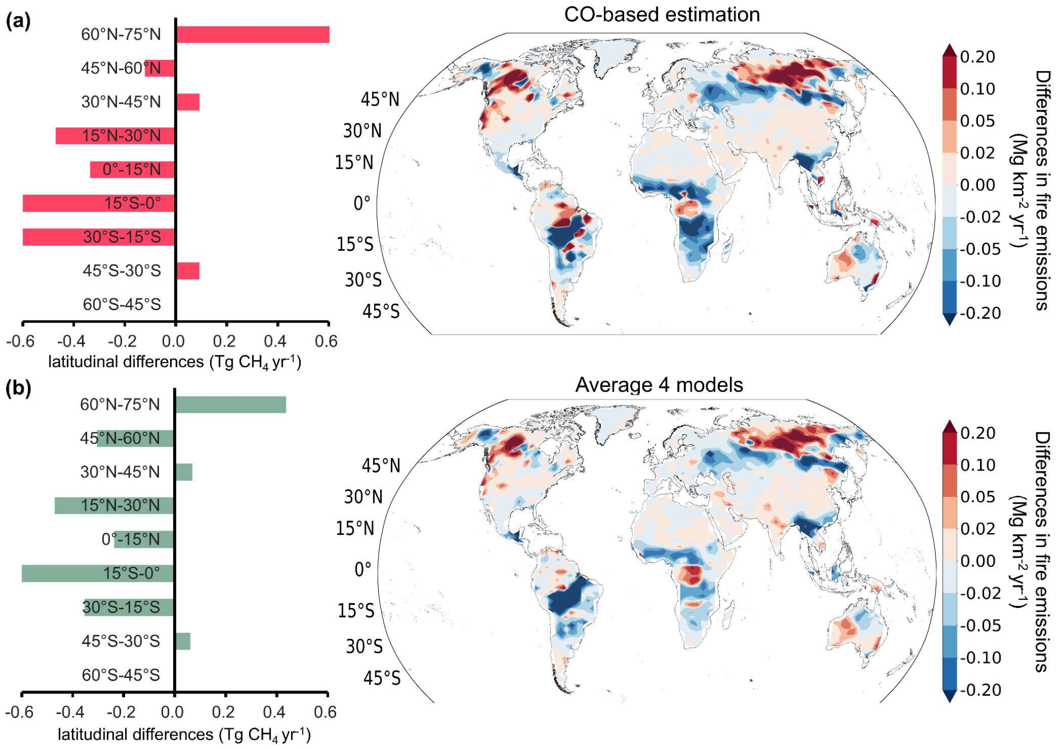

Supplementary Fig. 3: Differences in decadal annual average fire CH_4 emissions from 2003–2011 to 2012–2020. The maps show the 2003–2011 annual average subtracted from the 2012–2020 annual average at a horizontal resolution of 3.75° longitude×1.9° latitud

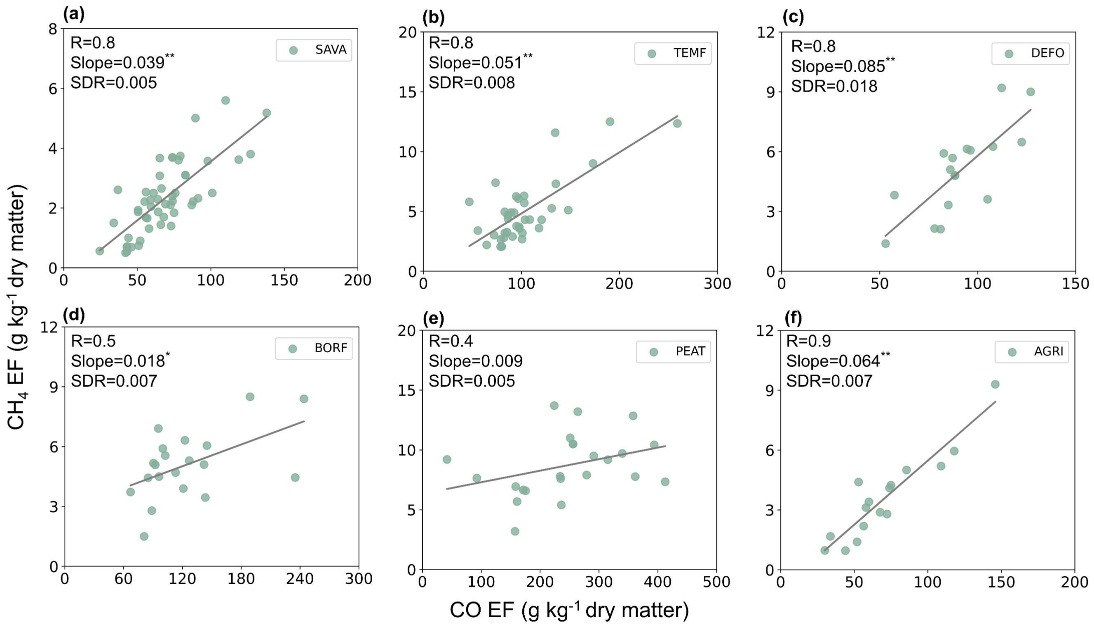

Supplementary Fig. 4: The 148 sets of field measurement data used for establishing the relationship between fire CO and fire CH_4 emission factors. This figure includes the measurement for fires over savanna (SAVA) (a), temperate forest (TEMF) (b), tropical forest (DEFO) (c), boreal forest (BORF) (d), peatland (PEAT) (e), and agriculture residues (AGRI) (f), sourced from Andreae et al.[21] and this study (Supplementary Table 3). The solid line denotes the fitted line, with significant trends marked by asterisks (* for P < 0.05 and ** for P < 0.01). R, Slope, and SDR represent Pearson's correlation coefficient, the regression line slope, and the standard deviation of residuals, respectively. These R are significant at the p < 0.05 level marked by asterisks (* for *p*< 0.05 and ** for *p* < 0.01).

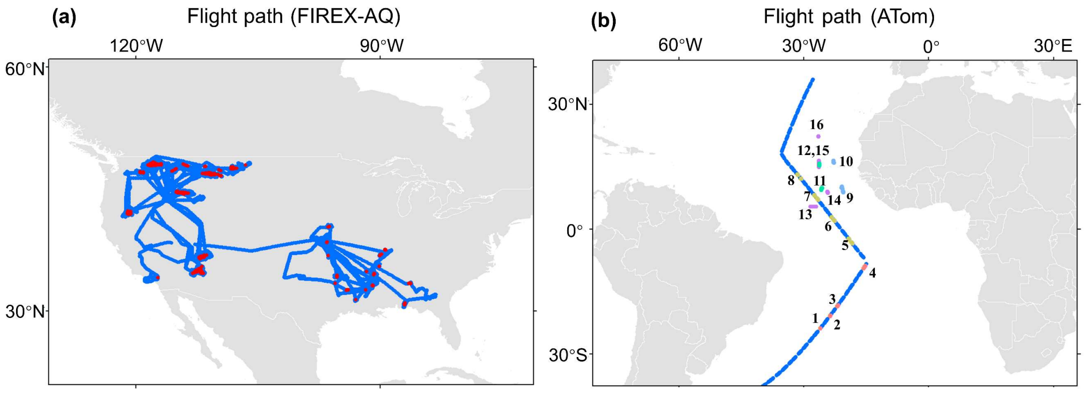

Supplementary Fig. 5: Flight tracks and fire plume locations. (a) The blue tracks represent the overall flight trajectories of FIREX-AQ from July 24 to September 5, 2019, with red dots indicating fire plume locations. (b) The blue track represents the flight trajectory of ATom on February 13 and 15, 2017, to the western region of Africa (55°S–35°N, 60°W–5°W), and the different colored dots represent fire plume locations listed in Supplementary Table 2.

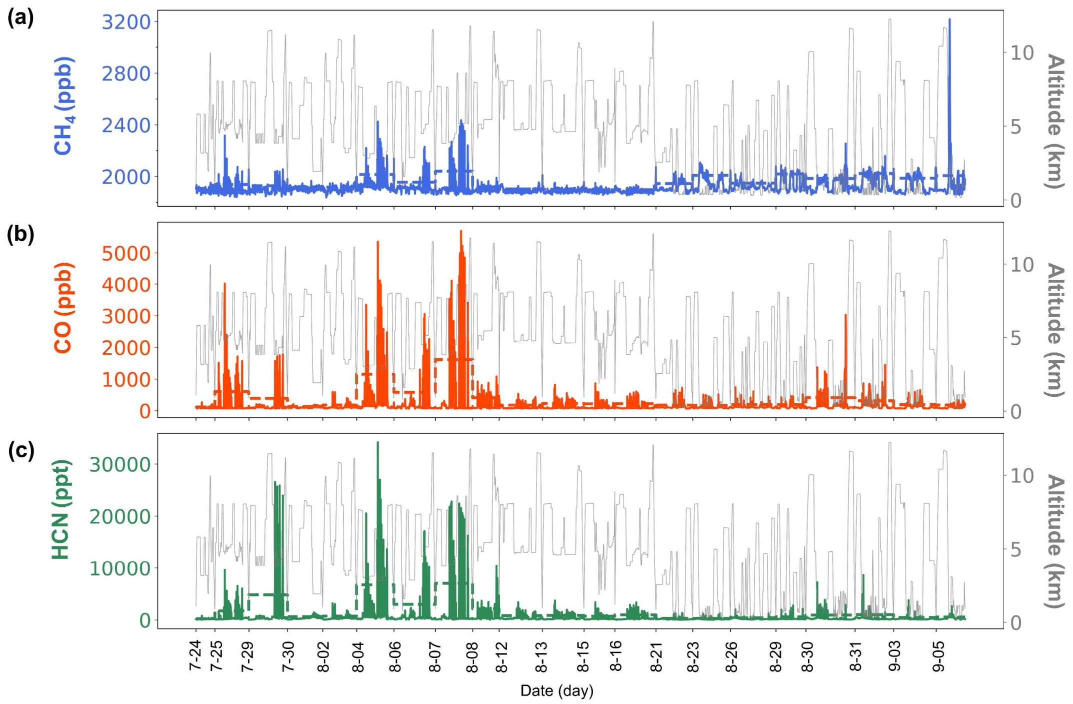

Supplementary Fig. 6: Time series of CH_4, CO, HCN, and altitude during FIREX-AQ (July 24 to September 5, 2019). Dashed horizontal lines show thresholds employed in fire plume identification.

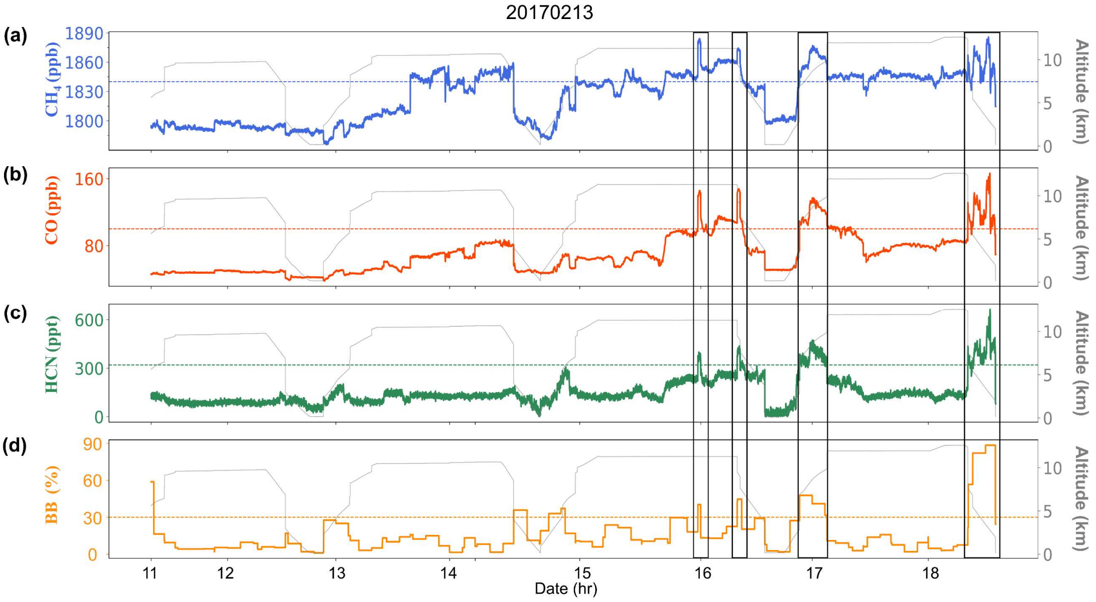

Supplementary Fig. 7: Time series of CH_4, CO, HCN, biomass burning (BB), and altitude during ATom on February 13, 2017. Fire plume intercept windows are indicated with black borders, and the dashed horizontal lines show thresholds employed in fire plume identification.

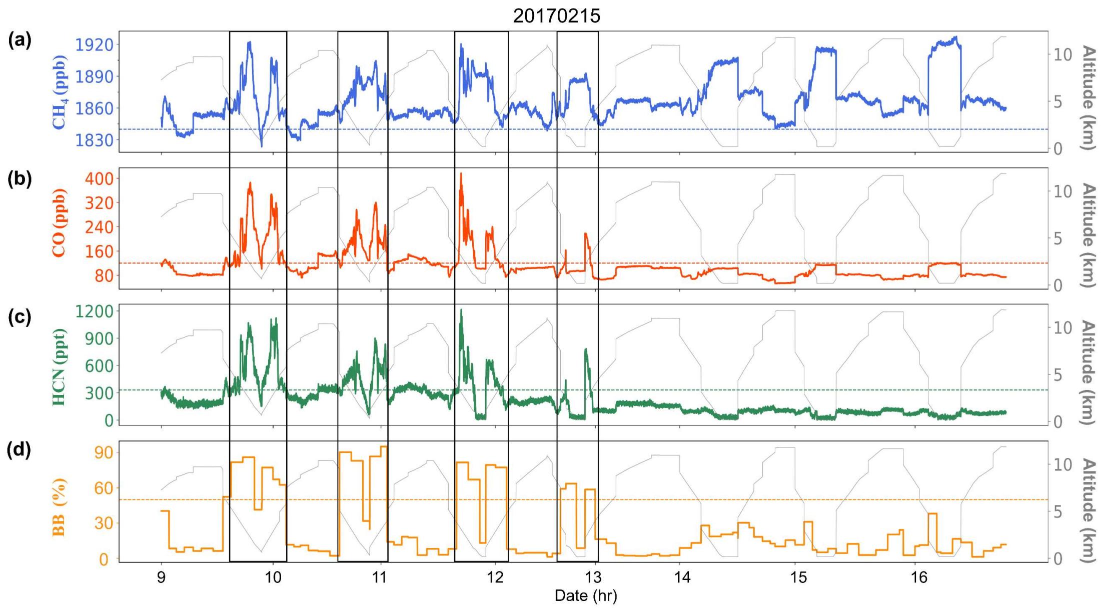

Supplementary Fig. 8: Time series of CH_4, CO, HCN, BB, and altitude during ATom on February 15, 2017. Fire plume intercept windows are indicated with black borders, and the dashed horizontal lines show thresholds employed in fire plume identification.

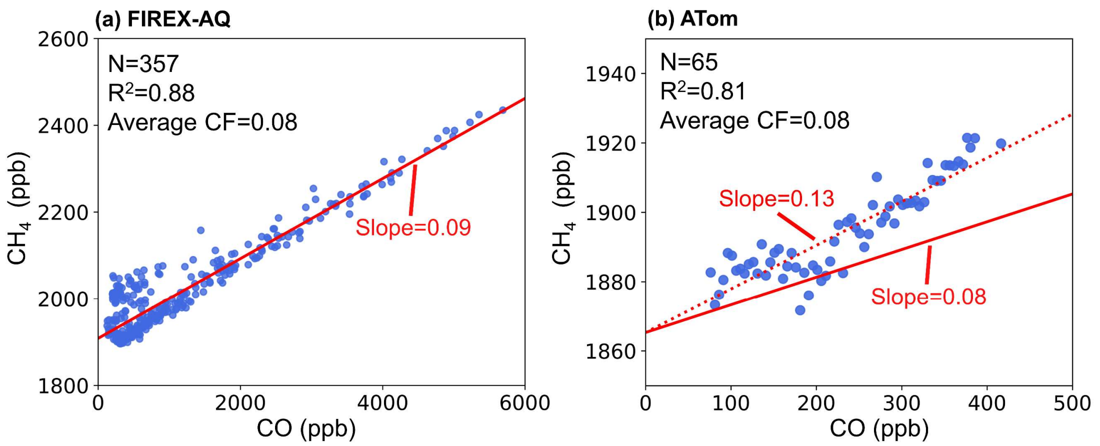

Supplementary Fig. 9: Evaluation of relationship between fire CH_4 and CO emission factors. (a) Airbone CH_4 and CO in-situ measurements of fire plume obtained during the FIREX-AQ flight campaign, represented by the blue dots. The red solid line correspond to the fitted line with a slope of 0.09, which represents the enhancment ratio of CH_4 to CO. (b) The graph illustrates the same concept as (a), but with CH_4 and CO observations from the ATom flight campaign. The red solid line represents the CH_4:CO enhancment ratio, accounting for the loss of each species during fire plume transport. The Average CF in (a) and (b) denotes the mean conversion factors are extracted from Supplementary Fig. 9.

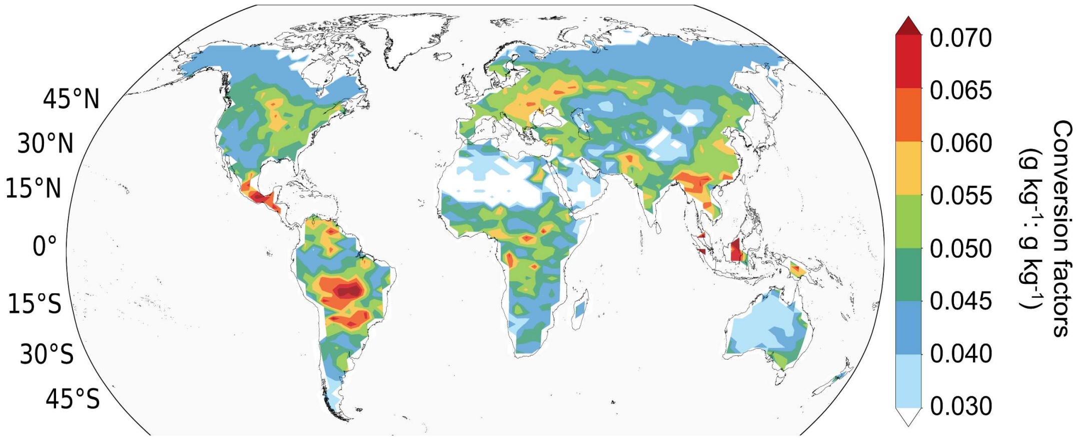

Supplementary Fig. 10: Spatial distributions of annual average conversion factors (2003-2020) derived from CH_4 :CO ratios and dry matter emissions in GFEDv4.1s (b). The spatial resolution of map shown here is 3.75° longitude and 1.9° latitude.

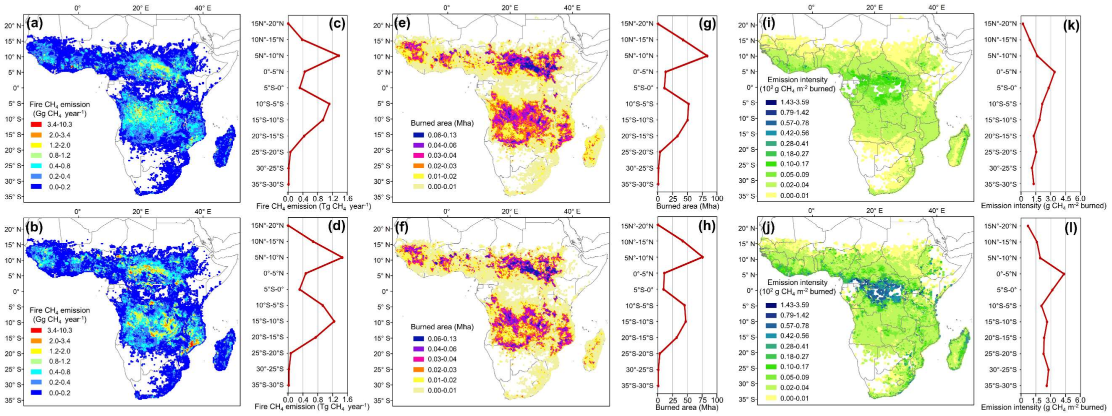

Supplementary Fig. 11: Fire CH_4 emission, burned area, and emission intensity in sub-Saharan Africa for 2016. Spatial distributions of fire CH_4 emission (a, b), burned area (e, f), and emission intensity(i, j) from GFEDv4.1s and M-500m at a resolution of 0.25° × 0.25°. Panels (c, d), (g, h), and (k, l) show latitudinal distributions of fire CH_4 emission, burned area, and emission intensity between 35°S and 20°N, where fire CH_4 emissions and burned area represent totals for each latitude band, and emission intensity is calculated as the total fire CH_4 emissions divided by the total burned area for the corresponding latitude band.

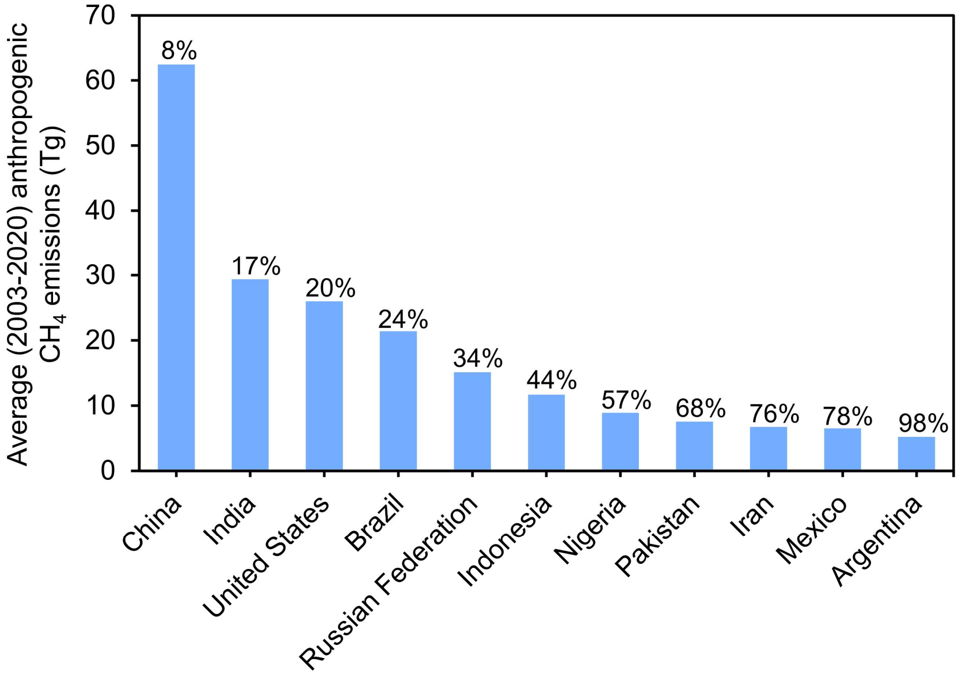

Supplementary Fig. 12: Annual average anthropogenic CH_4 emissions by country during 2003-2020 from EDGARv7.0[22]. The total difference (equivalent to 5.1 Tg yr^{-1}) between our results and the four models as a proportion of each country's total anthropogenic CH_4 emissions is indicated by the black numbers on top of the bars. . The annual average anthropogenic CH_4 emissions for other countries, which are lower than the average differences (~5.1 Tg yr^{-1}) between bottom-up fire emissions and CO inversion-based fire CH_4 emission estimates, are not displayed in this figure.

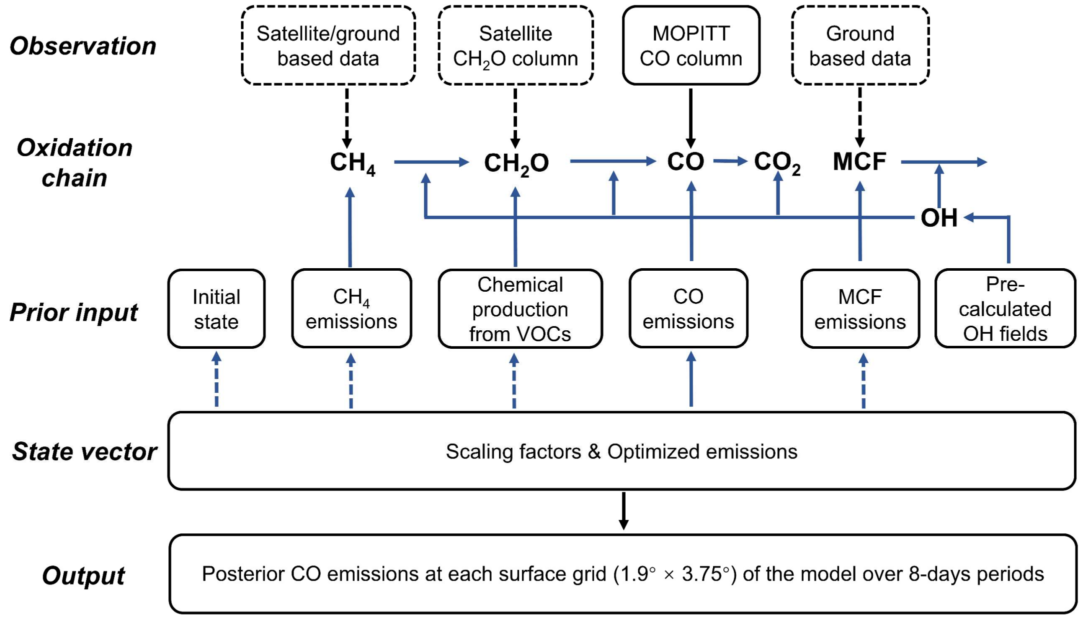

Supplementary Fig. 13: Modeling processes of the inversion method in this study. The CO column retrievals are derived from the MOPITT V9 product[23].The inversion system is capable of inferring surface flux variations for multiple species, including CO, CH_2O, CH_4, and MCF, from atmospheric measurements; however, this study only assimilates MOPITT CO data. Solid boxes represent the data and processes utilized in this study, while dashed boxes indicate those not applied

References

1. Pison, I., Bousquet, P., Chevallier, F., Szopa, S. & Hauglustaine, D. Multi-species inversion of CH_4, CO and H_2 emissions from surface measurements. *Atmos. Chem. Phys.* **9**, 5281–5297 (2009).

2.Stein O. et al.On the wintertime low bias of Northern Hemisphere carbon monoxide found in global model simulations. *Atmos. Chem. Phys.* **14**, 9295-9316 (2014).

3. Chevallier, F. et al. Inferring CO_2 sources and sinks from satellite observations: method and application to TOVS data. *J. Geophys. Res. Atmos.* **110**, D24309 (2005).

4. Chevallier F. et al. African CO emissions between years 2000 and 2006 as estimated from MOPITT observations. *Biogeosciences* **6**, 103–111 (2009).

5. Zheng, B. et al. Rapid decline in carbon monoxide emissions and export from East Asia between years 2005 and 2016. *Environ. Res. Lett.* **13**, 044007 (2018).

6. Zheng, B., Chevallier, F., Ciais, P., Yin, Y. & Wang, Y. On the Role of the Flaming to Smoldering Transition in the Seasonal Cycle of African Fire Emissions. *Geophys. Res. Lett.* **45**, 998–12,007 (2018)

7. Zheng, B. et al. Global atmospheric carbon monoxide budget 2000–2017 inferred from multi-species atmospheric inversions. *Earth Syst. Sci. Data* **11**, 1411–1436 (2019).

8.Wiedinmyer, C. et al. The Fire Inventory from NCAR version 2.5: an updated global fire emissions model for climate and chemistry applications. *Geosci. Model Dev.* **16**, 3873–3891 (2023).

9. Kaiser J.W. et al. Biomass burning emissions estimated with a global fire assimilation system based on observed fire radiative power. *Biogeosciences* **9**, 527-554 (2012).

10. Darmenov, A. & da Silva, A. M. The Quick Fire Emissions Dataset (QFED) - Documentation of versions 2.1, 2.2 and 2.4, NASA TM-2013-104606, **32**, 183 pp, (http://gmao.gsfc.nasa.gov/pubs/tm/) (2013).

11. van der Werf G.R. et al. Global fire emissions estimates during 1997–2016. *Earth Syst. Sci. Data* **9**, 697-720 (2017).

12. Chen, X. et al. HCOOH in the remote atmosphere: constraints from atmospheric tomography (ATom) airborne observations. *ACS Earth Space Chem.* **5**, 1436–1454 (2021)

13.Vernooij R. et al. Intraseasonal variability of greenhouse gas emission factors from biomass burning in the Brazilian Cerrado. *Biogeosciences* **18**, 1375-1393 (2021).

14.Frausto-Vicencio I. et al. Ground solar absorption observations of total column CO, CO2, CH4, and aerosol optical depth from California's Sequoia Lightning Complex Fire: emission factors and modified combustion efficiency at regional scales. *Atmos. Chem. Phys.* **23**, 4521-4543 (2023).

15.Wiggins E.B. et al. Boreal forest fire CO and CH4 emission factors derived from tower observations in Alaska during the extreme fire season of 2015. *Atmos. Chem. Phys.* **21**, 8557-8574 (2021).

16.Urbanski S.P. et al. Fuel layer specific pollutant emission factors for fire prone forest ecosystems of the western U.S. and Canada. *Atmospheric Environment: X* **16,**100188 (2022).

17.Yokelson R.J. et al. Tropical peat fire emissions: 2019 field measurements in Sumatra and Borneo and synthesis with previous studies. *Atmos. Chem. Phys.*, **22**, 10173-10194 (2022).

18.Lestari P. et al. Gaseous, particulate matter, carbonaceous compound, water-soluble ion, and trace metal emissions measured from 2019 peatland fires in Palangka Raya, Central Kalimantan. *Atmospheric Environment* **316,** 120171 (2024).

19.Hu Y. and Rein G. Development of gas signatures of smouldering peat wildfire from emission factors. *Int. J. Wildland Fire* **31**,1014-1032 (2022).

20.Watson J.G. et al. Gaseous, PM2.5 mass, and speciated emission factors from laboratory chamber peat combustion. *Atmos. Chem. Phys.* **19**,14173-14193 (2019).

21. Andreae, M. O. Emission of trace gases and aerosols from biomass burning—an updated assessment. *Atmos. Chem. Phys.* **19**, 8523–8546 (2019).

22. Crippa, M. et al. *GHG Emissions of All World Countries - 2021 Report*. Report No. 978-92-76-41547−3, (Publications Office of the European Union, 2021).

23. Deeter, M. et al. The MOPITT version 9 CO product: sampling enhancements and validation. *Atmos. Meas. Tech.* **15**, 2325–2344 (2022).
